# Constriction ring of the penis in a newborn infant: A rare form of amniotic band syndrome

**DOI:** 10.1002/ccr3.5618

**Published:** 2022-03-17

**Authors:** Yu Shan Ting, Renisha Paul Mukkam, Suresh Chandran

**Affiliations:** ^1^ 63751 Department of Neonatology KK Women's and Children's Hospital Singapore City Singapore; ^2^ Paediatrics Academic Clinical programme Yong Loo Lin School of Medicine National University of Singapore Singapore City Singapore; ^3^ Paediatrics Academic Clinical programme Lee Kong Chian School of Medicine Nanyang Technological University Singapore City Singapore; ^4^ Paediatrics Academic Clinical programme Duke‐NUS Medical School National University of Singapore Singapore City Singapore

**Keywords:** amniotic band, constriction ring syndrome, penile constriction ring, urethral obstruction

## Abstract

The amniotic band comprises disrupted amnion strands causing entrapment or entanglement of various fetal parts resulting in a spectrum of anomalies from digital band constriction or amputation to severe craniofacial/visceral defects and even fetal demise. We present a newborn infant with a rare, isolated ring constriction of the penis.

## Graphical abstract

Amniotic band syndrome comprises a highly variable spectrum of anomalies affecting the newborn infant's limbs, viscera, skull, and rarely genitals. Using a 3D ultrasound scan, band‐related fetal abnormalities can be diagnosed. Acquired constriction ring syndrome must be considered in the differential diagnosis of constriction lesions in older infants.

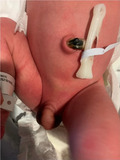

## 1 INTRODUCTION

Amniotic band syndrome (ABS), also known as constriction ring syndrome (CRS), was recognized as early as 300BC.^1^ The precise incidence of ABS is unclear but varies from 1 in 1200 to 1 in 15,000 live births. Bands are mesoblastic fibrous strings emanating from the amnion, causing disruption, deformation, and malformation of fetal parts.^2^ Fetal anomalies in ABS were classified into limb, craniofacial, and visceral defects by Seeds et al.^3^ More often, limb defects were reported due to entangling of the limbs by the floating amniotic bands resulting in digital to limb constrictions or even amputations.^4^ Major craniofacial presentations in ABS include acalvaria, acrania, anencephaly, encephalocele, facial clefts, nasal, and eye defects. Visceral defects vary from omphalocele, gastroschisis to limb‐body wall complex.^2^, ^3^ Reports of genital anomalies due to CRS are rare, and to the best of our knowledge, this is the second case report depicting a genital manifestation of CRS. In an asymptomatic infant, this clinical finding can be missed in the newborn examination as the skin over the shaft of the penis folds up in a non‐erectile state, masking the constriction ring.

## 2 CASE REPORT

A term well male infant was born by emergency cesarean section for decreased fetal movements and low amniotic fluid index (AFI). Parents were non‐consanguineous, and maternal serologies were normal. First and second trimester fetal ultrasound scans (USS) were unremarkable, and term USS showed AFI at 5th centile. Apgar scores were 9 at 1 and 5 minutes of life. He weighed 2694 g (28th centile), and his head circumference was 34 cm (68^th^ centile).

**FIGURE 1:**
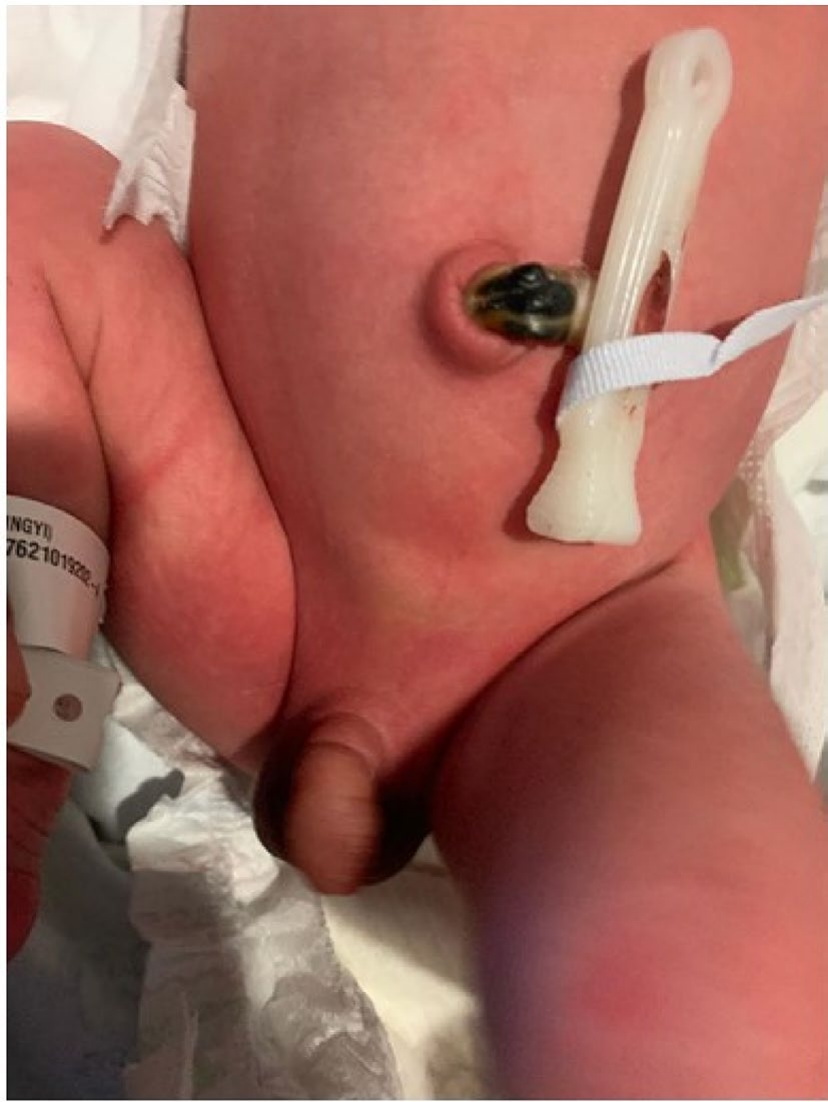
Hidden penile ring constriction in a non‐erectile state. The folds of skin over the shaft of the penis mask the ring constriction. Edema distal and proximal to the point of constriction visible

**FIGURE 2:**
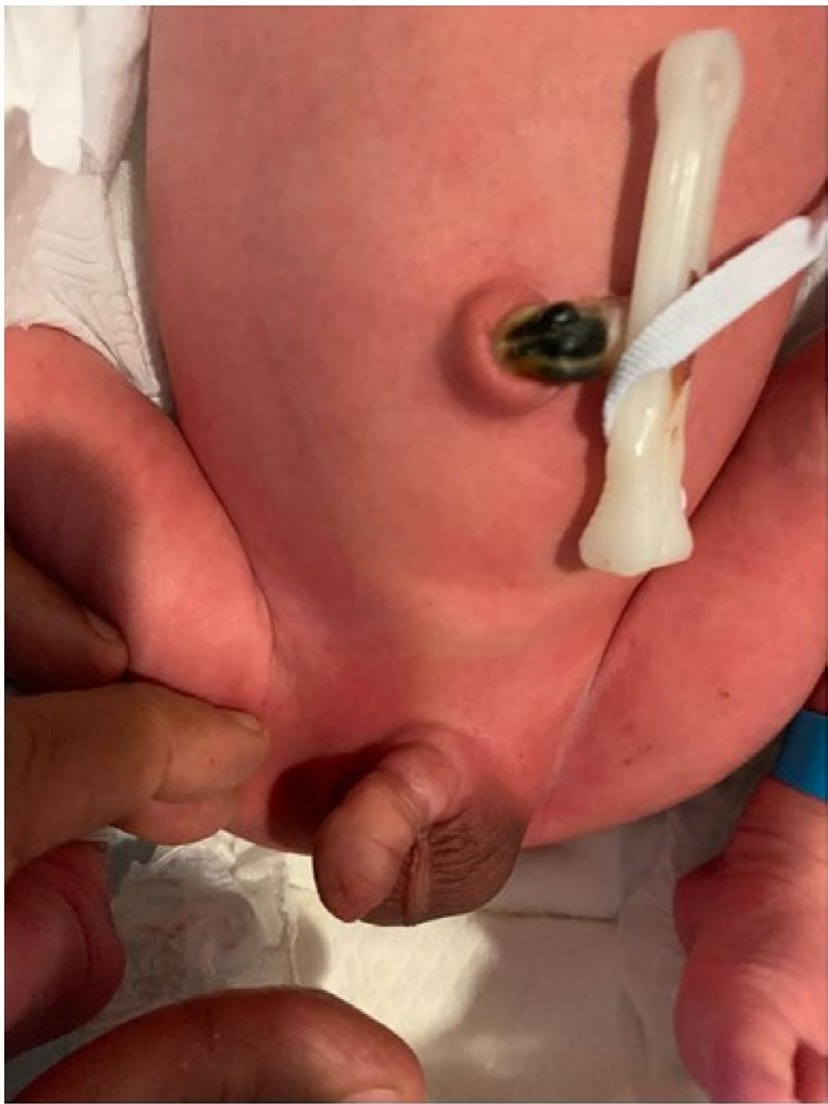
On stretching of skin over the shaft of the penis, the constriction ring is visible

His general physical examination was normal except for a constriction ring of scarred tissue at the lower third of the shaft of the penis, hidden beneath the folded skin (Figure 1). The penis measured 2.8 cm in length. The skin over the penis was edematous, both proximal and distal to the constriction ring, but more so distally. The constriction ring was scary and could slide over the shaft of the penis with some limitation (Figure 2). While examining, the neonatologist observed a normal penile erection on stimulation of genitalia and an adequate urinary stream. Testes were palpable in the well‐formed scrotum. The abdomen was soft, and the bladder was not palpable. No craniofacial defects or limb involvement was noted in our case.

An ultrasound scan of the kidneys, ureters, and bladder was unremarkable. Pediatric surgical opinion was sought, and the parents were counseled for follow‐up and needful surgical intervention if required with penile growth. He was discharged home on day 4 of life on full breastfeeds with advice for parental observation of the urinary stream.

He was followed up at 3 months in a combined clinic with the pediatric surgery team. There were no parental concerns. Edema over the penis remains the same. Otherwise, his growth and neurodevelopmental assessments were within normal limits.

## 3 DISCUSSION

We present a rare form of CRS affecting the shaft of the penis in a newborn infant. Most of the reported cases of CRS are sporadic, and no chromosomal anomalies were ever reported.^2^ Several theories related to the pathogenesis of ABS has been debated. George Streeter, in 1930 stated the possible etiology of CRS as germplasm defects, known as embryonic dysplasia theory (endogenous). Malfunction of embryonic placodes involved in the formation of organs and structures results in malformations.^5^

In 1961, Patterson used histologic evidence of the lack of mesodermal development in constriction bands, hypothesizing that these constrictions are abnormal skin creases. Constriction rings seen in limbs are classified according to Patterson criteria.^6^

Later, in 1965, Torpin reported that early amnion rupture sequence (exogenous theory) could explain the variety of presentations seen in fetuses affected by CRS. On rupture of amnion, the fetus lies outside the amniotic cavity. The ensuing oligohydramnios may aggravate the deformities seen in ABS. Ruptured amnion emanates mesoblastic fibrous strings from the chorionic side of amnion, which can entangle or entrap fetal parts, more often the limbs causing digital/limb amputation and less often major visceral defects and rarely involving the skull. Constriction of normally developing parts of the body can result in CRS, especially the extremities.^2^, ^4^ Severe malformations result from early and milder forms from late amnion rupture.^7^

Van Allen, in 1981, proposed the vascular disruption theory, where interference with the embryonic blood supply distorts the normal morphogenesis or damage the existing anatomic structure that may result in malformation or deformation.^8^

None of these theories could explain all the findings noted in ABS. Torpin's exogenous theory remains the most widely accepted to this day.

There was low AFI at term gestation and a milder form of CRS in the reported case, indicating late amnion rupture and supports exogenous theory by Torpin.

In a non‐erectile state, the penis lies on the scrotum, thigh, inguinal area, or abdominal wall in fetal life. On erection, the fetal penis is almost at right angles to that of the skin of the scrotum.^9^ It is known that in CRS, the more prominently protruding structures like the middle finger of the hand, being the most extended finger, is more frequently and severely affected than the other fingers.^10^ Similarly, the erectile state predisposes the shaft of the penis to get entrapped in the bands floating in the amniotic fluid.

To date, the only report of ABS involving the penis was reported by Chen et al.^11^ They described a newborn infant having a severe ring constriction of the fetal penile shaft causing urethral stricture leading to distal obstructive uropathy and Prune‐belly syndrome. There is no current evidence of vascular compromise of the penis or urethral obstruction due to the constriction ring in our reported case. However, with penile growth in puberty and adulthood, when the corpora cavernosa and corpus spongiosum grow, and the fibrotic skin over the penis remains tight at the constriction point, there is a potential for progressive urethral obstruction or priapism.

Acquired constriction ring syndrome (ACRS), also known as hair‐thread tourniquet syndrome, is characterized by circumferential constriction of any appendage (toe, genital, etc.) by human hair or fiber/thread from clothing. ACRS is thought to be congenital with the source of hair from early fetal shedding or separated hair due to fetal movements. The constriction ring of an appendage can occur after birth following entangling of digits or appendages on exposure to environmental hair or fiber. ACRS should be considered in the differential diagnosis of constriction lesions in infants, as 80% of cases are reported in <2 months old.^12^

Prenatal diagnosis of ABS remains challenging. Seeds et al. recommended ruling out ABS when gross fetal anomalies are detected on fetal sonography.^3^ Fetal imaging using 3D USS showed the accuracy of identifying the spatial relationship between bands and the body of the fetus, enabling diagnosis of ABS in early gestation.^13^

## 4 CONCLUSION

The spectrum of possible anomalies and many combinations lead to a highly variable presentation of ABS; hence, this condition is often underdiagnosed. ABS syndrome is a clinical diagnosis. It is worthwhile for pediatricians caring for newborn infants to be aware of these rare band‐related genital defects and continue long‐term follow‐ups to avoid urogenital morbidity.

## CONFLICT OF INTEREST

The authors declare that there are no conflicts of interest.

## AUTHOR CONTRIBUTIONS

Author 1 wrote the first draft of the manuscript—Introduction and case report. She also did the language edit. Author 2 involved in diagnosis, management of the case, and revision of the introduction and case report part of the manuscript. Author 3 involved in diagnosis, management of the case, follow‐up care and wrote the discussion and abstract. Edited the whole manuscript and added suitable references.

## ETHICAL APPROVAL

Case reports are exempted from obtaining ethics approval by Internal review Board, SingHealth, Singapore.

## CONSENT

Written informed consent was obtained from the patient to publish this report in accordance with the journal's patient consent policy.

## Data Availability

Data available at request from the corresponding author.
